# Field and laboratory comparative evaluation of rapid malaria diagnostic tests versus traditional and molecular techniques in India

**DOI:** 10.1186/1475-2875-9-191

**Published:** 2010-07-05

**Authors:** Neeru Singh, Man M Shukla, Mohan K Shukla, Rajiv K Mehra, Shweta Sharma, Praveen K Bharti, Mrigendra P Singh, Ajay Singh, Arunachalam Gunasekar

**Affiliations:** 1 Regional Medical Research Centre for Tribals (ICMR), RMRCT Campus, Nagpur Road, Jabalpur 482003, Madhya Pradesh, India; 2 National Institute of Malaria Research (ICMR), Field Station, RMRCT Campus, Nagpur Road, Jabalpur 482003, Madhya Pradesh, India; 3 World Health Organization India Office, 534, "A" Wing, Nirman Bhawan, Maulana Azad Road, New Delhi 110011, India

## Abstract

**Background:**

Malaria presents a diagnostic challenge in most tropical countries. Microscopy remains the gold standard for diagnosing malaria infections in clinical practice and research. However, microscopy is labour intensive, requires significant skills and time, which causes therapeutic delays. The objective of obtaining result quickly from the examination of blood samples from patients with suspected malaria is now made possible with the introduction of rapid malaria diagnostic tests (RDTs). Several RDTs are available, which are fast, reliable and simple to use and can detect *Plasmodium falciparum *and non-falciparum infections or both. A study was conducted in tribal areas of central India to measure the overall performance of several RDTs for diagnosis of *P. falciparum *and non-falciparum infections in comparison with traditional and molecular techniques. Such data will be used to guide procurement decisions of policy makers and programme managers.

**Methods:**

Five commercially available RDTs were tested simultaneously in field in parallel with peripheral blood smears in outbreak-affected areas. The evaluation is designed to provide comparative data on the performance of each RDT. In addition, molecular method i.e. polymerase chain reaction (PCR) was also carried out to compare all three methods.

**Results:**

A total of 372 patients with a clinical suspicion of malaria from Bajag Primary Health Centre (PHC) of district Dindori and Satanwada PHC of district Shivpuri attending the field clinics of Regional Medical Research Centre were included in the study. The analysis revealed that the First Response Malaria Antigen pLDH/HRP2 combo test was 94.7% sensitive (95% CI 89.5-97.7) and 69.9% specific (95% CI 63.6-75.6) for *P. falciparum*. However, for non-falciparum infections (*Plasmodium vivax*) the test was 84.2% sensitive (95% CI 72.1-92.5) and 96.5% specific (95% CI 93.8-98.2). The Parascreen represented a good alternative. All other RDTs were relatively less sensitive for both *P. falciparum *and non-falciparum infections.

**Conclusions:**

The results in this study show comparative performance between microscopy, various RDTs and PCR. Despite some inherent limitation in the five RDTs tested, First Response clearly has an advantage over other RDTs. The results suggest that RDTs could play and will play an important role in malaria diagnosis.

## Background

Malaria is a disease of global importance that results in 300 - 660 million cases annually and an estimated 2.2 billion people are at risk of infection [1]. Of the 2.5 million reported cases in the South East Asia, India alone contributes about 70% of the total malaria cases [2]. Currently, 80.5% of the 109 billion population of India lives in malaria risk areas [3]. Malaria presents a diagnostic challenge in most resource poor areas where malaria is endemic. In such areas malaria diagnosis is often made only on the basis of clinical symptoms although this is alarmingly inaccurate [4]. The role of the laboratory diagnosis of malaria is primarily to support clinical care [5]. The traditional method for the detection of the malaria parasite is the examination of thick and thin blood smears by microscopy. The shortcomings of microscopy for malaria diagnosis are well known [6,7]. These diagnostic limitations affect medical care provided, as malaria is a potentially fatal disease, usually curable if diagnosed quickly [8]. The World Health Organization has recognized the urgent need for simple and cost effective diagnostic tests for malaria to overcome the deficiencies of both light microscopy and clinical diagnosis [9]. The need for a simple, sensitive diagnostic test has led to the development of rapid diagnostic tests (RDTs) among other alternative techniques. Initially the use of RDTs met stiff resistance by the malaria community because of its cost. However, a number of reports from policy makers have acknowledged that RDTs may have their place because expert microscopy in malaria-endemic countries is hard to establish and cost of RDTs has been greatly reduced [10]. Further, the recently introduced artemisinin based combination therapy (ACT) is given to patients only when the diagnosis has been confirmed parasitologically. However, providing parasitological results at all levels of health care presents a serious challenge. Expanding the use of blood slide microscopy is a possible solution but the cost and logistic challenges in remote area restricts the use of microscopy to hospitals and well-equipped laboratories. It takes great skill and years of experience to learn to accurately read a malaria slide. The use of RDTs for *Plasmodium falciparum *malaria is being implemented by National Vector Borne Disease Control Programme (NVBDCP) to improve diagnostic efficiency in peripheral health care settings in central India. Further, although *P. falciparum *causes the most severe disease, recent reports of significant morbidity and drug resistance in *Plasmodium vivax *infections are generating new interest in *P. vivax *[11,12]. The first generation RDT tests were specific for *P. falciparum *[13-15], but the development of new rapid tests by including a Pan-malaria test line allowed detection of non-falciparum infections also [16,17]. Subsequently increasing numbers of similar products have been developed [7,18,19].

We undertook a study on evaluation to assess the performance of commercially available malaria RDTs in comparison with microscopy and polymerase chain reaction (PCR) in an area where both *P. falciparum *and *P. vivax *are co-endemic. This would allow direct product comparisons that would assist the policy makers and programme managers in taking procurement decisions and would ultimately encourage improvement in the quality of manufacturing. Five RDTs evaluated for this purpose were selected on the basis of two main criteria i.e. tests detecting both *P. falciparum *and non-falciparum infections and commercial availability.

## Methods

### Study area

**Figure 1:**
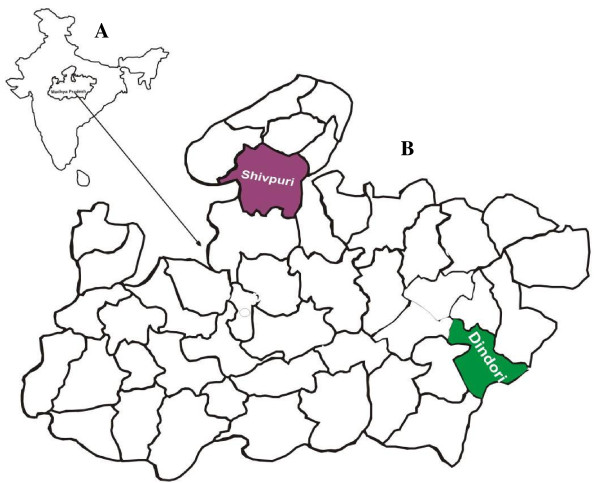
**(A) Map of India showing Madhya Pradesh, (B) Shivpuri and Dindori district**.

The study was conducted in 10 villages of Bajag Primary Health Centre of district Dindori (22°57' N latitude and 81°41' E longitude) between August to December, 2009. Dindori is a highly malarious district in Madhya Pradesh (Figure 1) contributing 12% of malaria in the state while its population is only 1% of state [20]. The villages of Bajag PHC are very remote, forested and inaccessible for 4-6 months during rainy season. The average annual rainfall is 1,400 mm. The inhabitants of these villages are ethnic group of Baiga primitive tribe. They are very poorly clothed and have immense faith in sorcery and witchcraft. There is no public transport system and health facilities are non-existent. *Plasmodium falciparum *is the predominant infection. The RDT evaluation was also carried out in 10 villages of Satanwada Primary Health Centre, District Shivpuri (25°4' N latitude and 77°44' E longitude). The inhabitants of the villages are ethnic group of Saharia primitive tribe who live in small one room hutments which are very overcrowded and unhygienic. *Plasmodium vivax *is the predominant infection in this area unlike Bajag PHC. The study area is hot, dry and the average annual rainfall is 875 mm.

### Study design

All patients irrespective of their age and sex except pregnant women presenting at the field clinic with a clinical suspicion of malaria were included in the study after taking written informed consent. This study was approved by the ethics committee of the Regional Medical Research Centre (ICMR).

Demographic and clinical information was recorded from each patient and all five RDTs were tested simultaneously in field in parallel with peripheral blood smear for microscopic examination in the laboratory. Before the initiation of the study, a one-day workshop was organized to provide training in blood collection from finger prick, test procedure for each RDT and interpretation of the test results as per manufacturer's instructions. The results of each RDT were recorded between 15-30 minutes as per the manufacturer's instructions. 2-3 drops of finger prick blood samples were also collected in heparinised tube/filter paper for PCR to be conducted in the laboratory.

The five RDTs used in this study are - Parascreen Device (rapid test for malaria Pan/Pf), Falcivax Device (rapid test for malaria Pv/Pf), Malascan Device (rapid test for malaria Pf/Pan) (all from Zephyer Biomedicals Goa), ParaHIT Total (rapid test for Pf & Pan Malaria species) (SPAN Diagnostics Ltd, Surat) and First Response Malaria Antigen Combo Card test (pLDH/HRP2) (Premier medical corporation Mumbai). These RDTs were provided by their manufacturers for the evaluation. The detailed characteristics of each test are shown in Table 1.

**Table 1:** Characteristics of evaluated rapid malaria tests

		Prascreen Deviice (Pan/Pf)	Malascan Device (Pf/Pan)	Falcivax (Pv/Pf)	Frist Response Malaria pLDH/HRP2 combo	Para HIT Total
**Plasmodium species targeted (F= P.*falciparum*V = *P.vivax*P = PAN)**		P,F	F,P	F,V	F,P	F,P
**Target Antigen**		HRP2/pLDH	HRP2/Aldolase	HRP2/Vivax specific pLDH	HRP2/pLDH	HRP2/Aldolase/pLDH
**Format**		Cassette	Cassette	Cassette	Cassette	Dipstick
**Sequence and type of bound antibody**	**C**	√	√	√	√	√
	**T_1_**	pLDH	Aldolase	pLDH	pLDH	**Aldolase/**pLDH
	**T_2_**	HRP2	HRP2	HRP2	HRP2	HRP2
**Required Volume (μ) of whole blood**		5	5	5	5	8
**Buffer Volume**		4 Drops	4 Drops	4 Drops	2 Drops	4 Drops
**Intermediate step**		-	-	-	-	Buffer into tube blood on stick, stick on tube
**Time to results (mins)**		15	15	15	20	15
**Maximum Reading time (mins)**		30	30	30	-	30

All RDTs were tested by two Research Assistants to minimize variability. The blood films were examined by an experienced microscopist in the laboratory without reference to the results of RDTs and clinical history of patient. The results of both microscopy and RDTs were matched by an independent expert. A slide was considered positive if atleast one asexual form of parasite was detected in 100 microscopic fields in thick blood film. Blood parasite density was determined from the thick films by counting the number of parasites against 200 white blood cells (WBC) and assuming that each subject had 8000 white blood cells/μl of blood. All negative slides that test positive on the RDT/PCR or all positive slides that test negative on the RDT/PCR were re-examined by another expert technician blinded to the results of microscopy, RDT/PCR and clinical status of the patients. The PCR was performed blind on coded samples by an independent Research Assistant unaware of clinical status of patients, result of RDTs and microscopic examination. Every person positive for falciparum malaria by RDT or by microscopy was treated with a combination of artesunate and sulphadoxine-pyremethamine (ACT) or with chloroquine (CQ) if RDT/microscopy showed non-falciparum infection.

For testing temperature stability of the tests, RDTs were stored at 25°C on receipt in the study sites, then allocated to separate groups for storage at 35°C & 45°C for 90 days, at 60°C for 48 hours, and at -10°C for 60 minutes before testing [21]. At the start of the study, the incubators were stabilized at the required temperature for three days before the RDTs to be tested were placed inside. RDTs were removed from storage to reach room temperature for 2 hours before testing and comparisons were made with control RDTs kept at 25°C until use and with microscopy. The malaria RDTs used in this study were from single lots of commercially available products.

### Polymerase chain reaction

The DNA was isolated from the blood by using the commercially available DNA extraction kit (Bio Basic Inc) as per manufacturer protocol and also from archive blood spots by Tris-EDTA (TE) buffer method. PCR for the identification of malaria parasite was performed following the standard methods [22].

### Data analysis

Results of the RDT and microscopy examination were recorded on separate forms. After double key data entry, the database was rechecked for all inconsistent entries and errors were corrected. Data were then analysed using STATA 8.2 (StataCorp, College Station Texas, USA). The figures for sensitivity, specificity, predictive values, accuracy, the area under the receiver operator characteristic curve (AUC) and the likelihood ratios were calculated using the 'diagt' command in Stata [23]. All estimated parameters are detailed with a 95% Confidence Interval (CI) unless stated otherwise.

## Results

**Figure 2:**
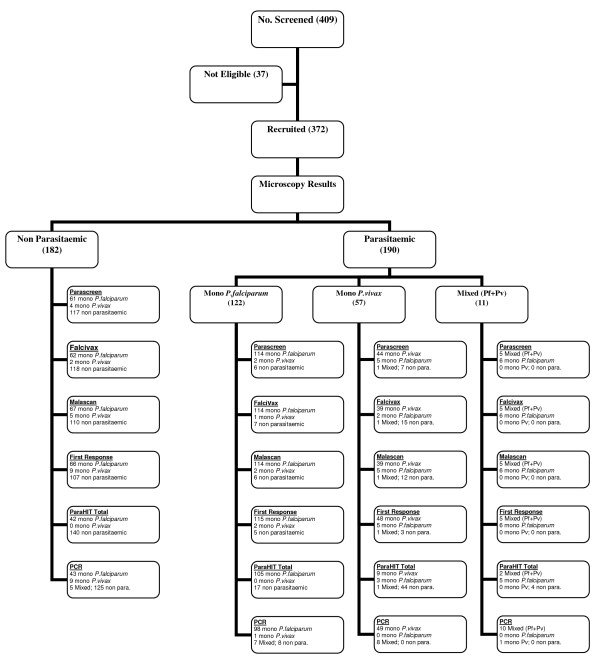
**Total field clinic attendance and patients recruited for malaria screening by Rapid Diagnostic Tests, Microscopy and PCR**.

During the study period, 409 patients (age 1 to 69 years) attended the two sites 236 patients (57.7%) were screened at Dindori and 173 (42.2%) were screened at Shivpuri (mean age 15.45 ± 14.15). 37 patients (9%) were excluded as not fulfilling the study enrolment criteria due to recent anti-malarial intake. 372 patients were eligible and all these patients were recruited (mean age 15.03 ± 14.07). All recruited patients were tested by microscopy, RDT and PCR (Figure 2).

A total of 190 (51.1%) were found infected by microscopy, 57 (15.3%) with *P. vivax*, 122 with *P. falciparum *(32.8%) and 11 (3%) with both *P. vivax *and *P. falciparum*. The overall sensitivity and specificity of First Response for malaria was 95.8 and 58.8%, Parascreen 93.2 and 64.3%, Malascan 90.5 and 60.4%, Falcivax 88.4 and 64.8% and by ParaHIT Total 65.8 and 76.9% respectively (Table 2). The highest sensitivity was recorded by First Response and was considered here as reference test for comparing the sensitivity and specificity of other RDTs. Parascreen was found to be 41% less sensitive than First Response (OR 0.59, 95%CI 0.24 - 1.48), though, this difference was not significant statistically (P > 0.05). Falcivax was 67% less sensitive (OR 0.33, 95%CI 0.14 - 0.78) and Malascan was 58% less sensitive (OR 0.42, 95%CI 0.18 - 1.00) than First Response and these differences were significant statistically (P < 0.05). ParaHIT Total showed lowest sensitivity (OR 0.08, 95% CI 0.04 - 0.19) and highly significant statistically (P < 0.0001). Thus, Parascreen was the first alternative to First Response. Similarly, further analysis showed that the specificity of Parascreen, Falcivax and Malascan were 1.07 to 1.26 times more when compared to First Response which is not significant (P > 0.05). However, specificity of ParaHIT Total was more than 2 times when compared to the First Response (OR 2.34, 95% CI 1.47 - 3.71), which was highly significant statistically.

**Table 2:** Comparative performance of Rapid Diagnostic Test kits (Parascreen, Falcivax, Malascan, First Response and ParaHIT Total) with traditional light microscopy and Polymerase Chain Reaction (PCR) for diagnosis of Malaria

	Parascreen	Falcivax	Malascan	First Response	ParaHIT Total
**Light Microscopy as Gold Standard (Reference)**					

Sensitivity	93.2	88.4	90.5	95.8	65.8
(95% CI)	(88.6-96.3)	(83.0-92.6)	(85.4-94.3)	(91.9-98.2)	(58.6-72.5)
Specificity	64.3	64.8	60.4	58.8	76.9
(95% CI)	(56.9-71.2)	(57.4-71.8)	(52.9-67.6)	(51.3-66.0)	(70.1-82.8)
Positive Likelihood Ratio	2.6	2.5	2.3	2.3	2.9
(95% CI)	(2.1-3.2)	(2.1-3.1)	(1.9-2.8)	(1.9-2.8)	(2.2-3.8)
Negative Likelihood Ratio	0.1	0.2	0.2	0.1	0.44
(95% CI)	(0.1-0.2)	(0.1-0.3)	(0.1-0.3)	(0.04-0.14)	(0.36-0.55)
Positive Predictive Value	73.1	72.4	70.5	70.8	74.9
(95% CI)	(67.1-78.6)	(67.2-78.1)	(64.3-76.1)	(64.8-76.3)	(67.6-81.2)
Negative Predictive Value	90.0	84.3	85.9	93.0	68.3
(95% CI)	(83.5-94.6)	(77.2-89.9)	(78.7-91.4)	(86.8-96.9)	(61.4-74.6)
Percentage Agreement (Accuracy)	79.0	76.9	75.8	77.7	71.2
Kappa	0.58	0.53	0.51	0.55	0.43

**PCR as Gold Standard (Reference)**					

Sensitivity	86.6	83.1	85.7	89.2	61.0
(95% CI)	(81.5-90.7)	(77.7-87.7)	(80.5-90.0)	(84.4-92.9)	(54.4-67.4)
Specificity	73.7	75.2	70.7	67.7	85.0
(95% CI)	(65.3-80.9)	(67.0-82.3)	(62.2-78.2)	(59.0-75.5)	(77.7-90.6)
Positive Likelihood Ratio	3.3	3.4	2.9	2.8	4.1
(95% CI)	(2.5-4.4)	(2.5-4.5)	(2.2-3.8)	(2.2-3.5)	(2.7-6.2)
Negative Likelihood Ratio	0.2	0.2	0.2	0.2	0.46
(95% CI)	(0.1-0.3)	(0.2-0.3)	(0.1-0.3)	(0.11-0.24)	(0.38-0.55)
Positive Predictive Value	85.1	85.3	83.5	82.7	87.6
(95% CI)	(79.9-89.4)	(80.0-89.7)	(78.2-88.0)	(77.5-87.2)	(81.5-92.2)
Negative Predictive Value	76.0	71.9	74.0	78.3	55.7
(95% CI)	(67.7-83.1)	(63.7-79.2)	(65.5-81.4)	(69.6-85.4)	(48.5-62.6)
Percentage Agreement (Accuracy)	81.9	80.2	80.2	81.3	69.8
Kappa	0.61	0.58	0.57	0.59	0.41

The species wise analysis revealed that the sensitivity for *P. falciparum *was highest (94.7%) by First Response (Table 3), while lowest for ParaHIT Total, (84.2%). The specificity for *P. falciparum *was highest (80.8%) by ParaHIT Total while lowest 69.5% by Malascan. The positive predictive value (PPV) for *P. falciparum *was highest by ParaHIT Total (70.9%) while lowest by Malascan (63.1%). The negative predictive value (NPV) was highest (96.0%) by First Response while lowest (90.2%) by ParaHIT Total. The false positive rate for *P. falciparum *was highest (30.5%) by Malascan (73/239) while lowest (19.2%) by ParaHIT Total (46/239). Like wise the false negative rate for *P. falciparum *was highest (15.8%) by ParaHIT Total (21/133) and lowest (5.3%) by First Response (7/133). When PCR was used as a reference standard the corresponding values for sensitivity, specificity, PPV, NPV and accuracy for each RDT are shown in Table 3.

**Table 3:** Comparative performance of Rapid Diagnostic Test kits (Parascreen, Falcivax, Malascan, First Response and ParaHIT Total) with traditional light microscopy and Polymerase Chain Reaction (PCR) for diagnosis of *P.falciparum*

	Parascreen	Falcivax	Malascan	First Response	ParaHIT Total
**Light Microscopy as Gold Standard (Reference)**					

Sensitivity	94.0	94.0	94.0	94.7	84.2
(95% CI)	(88.52-97.4)	(88.5-97.4)	(88.5-97.4)	(89.5-97.7)	(76.9-90.0)
Specificity	72.0	72.8	69.5	69.9	80.8
(95% CI)	(65.8-77.6)	(66.7-78.3)	(63.2-75.2)	(63.6-75.6)	(75.2-85.6)
Positive Likelihood Ratio	3.4	3.46	3.08	3.14	4.38
(95% CI)	(2.7-4.1)	(2.80-4.27)	(2.53-3.74)	(2.58-3.83)	(3.34-5.73)
Negative Likelihood Ratio	0.08	0.08	0.09	0.08	0.20
(95% CI)	(0.04-0.16)	(0.04-0.16)	(0.04-0.17)	(0.04-0.16)	(0.13-0.29)
Positive Predictive Value	65.1	65.8	63.1	63.6	70.9
(95% CI)	(57.9-71.8)	(58.6-72.5)	(56.0-69.9)	(56.5-70.3)	(63.1-77.8)
Negative Predictive Value	95.6	95.6	95.4	96.0	90.2
(95% CI)	(91.4-98.1)	(91.5-98.1)	(91.1-98.0)	(91.9-98.4)	(85.4-93.8)
Percentage Agreement (Accuracy)	79.8	80.4	78.2	78.8	82.0
Kappa	0.60	0.61	0.57	0.58	0.62

**PCR as Gold Standard (Reference)**					

Sensitivity	83.6	84.8	84.8	83.6	73.7
(95% CI)	(77.2-88.8)	(78.5-89.8)	(78.5-89.8)	(77.2-88.8)	(66.4-80.1)
Specificity	78.2	80.3	76.2	75.6	86.5
(95% CI)	(71.7-83.8)	(74.0-85.7)	(69.5-82.0)	(69.0-81.5)	(80.9-91.0)
Positive Likelihood Ratio	3.84	4.31	3.56	3.43	5.47
(95% CI)	(2.92-5.06)	(3.22-5.77)	(2.74-4.61)	(2.65-4.44)	(3.78-7.91)
Negative Likelihood Ratio	0.21	0.19	0.20	0.22	0.30
(95% CI)	(0.15-0.30)	(0.13-0.27)	(0.14-0.29)	(0.15-0.31)	(0.24-0.39)
Positive Predictive Value	77.3	79.2	75.9	75.3	82.9
(95% CI)	(70.62-83.1)	(72.6-84.9)	(69.2-89.8)	(68.5-81.2)	(76.0-88.5)
Negative Predictive Value	84.4	85.6	85.0	83.9	78.8
(95% CI)	(78.2-89.3)	(79.7-90.4)	(78.8-89.9)	(77.6-89.0)	(72.6-84.1)
Percentage Agreement (Accuracy)	80.8	82.4	80.2	79.4	80.5
Kappa	0.62	0.65	0.61	0.59	0.61

**Figure 3:**
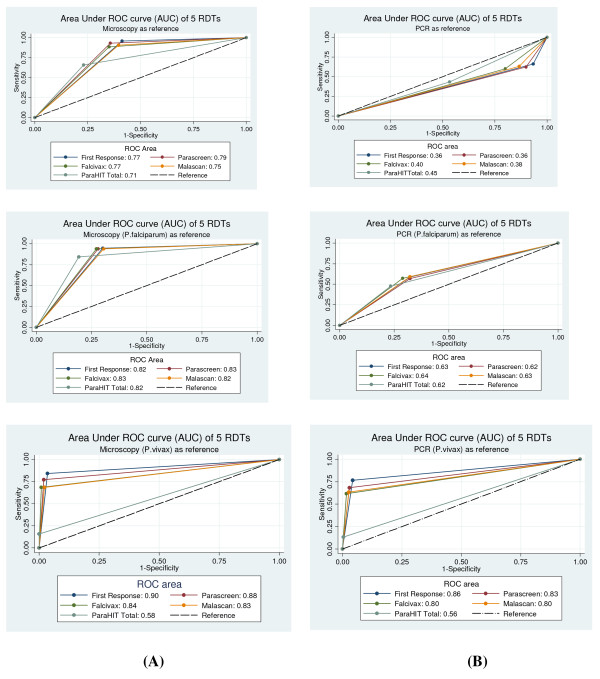
**Area under Receiver Operating Characteristic (ROC) curve (AUC) of 5 RDTs Vs light microscopy (A) and PCR (B) as reference test**.

For non-falciparum infections i.e. *P. vivax *the sensitivity of the test when compared with microscopy was 84.2% by First Response, while only 15.8% by ParaHIT Total (Table 4). Specificity of the test was 100% by ParaHIT Total and 96.5% by First Response. Similarly, PPV was highest (100%) for ParaHIT Total while lowest (81.4%) for First Response. On the contrary, NPV was highest for First Response (97.1%) while lowest (86.8%) by ParaHIT Total. False positive rate for *P. vivax *was highest (3.5%) by First Response (11/315) and none by ParaHIT Total (0/315). On the contrary, false negative rate for *P. vivax *was lowest (15.8%) by First Response (9/57) and highest (84.2%) by ParaHIT Total (48/57). The values of sensitivity, specificity, PPV, NPV and accuracy using PCR as reference standard are shown in Table 4. Area under Receiver Operating Characteristic (ROC) curve (AUC) of five RDTs *vs *microscopy was computed for diagnosing malaria, *P. falciparum *and *P. vivax *(Figure 3) The AUC of different RDTs were significantly different for malaria (χ^2 ^= 18.21, P < 0.001), and for *P. vivax *(χ^2 ^= 108.29, P < 0.0001) but not significant for *P. falciparum *(χ^2 ^= 8.47, P > 0.05).

**Table 4:** Comparative performance of Rapid Diagnostic Test kits (Parascreen, Falcivax, Malascan, First Response and ParaHIT Total) with traditional light microscopy and Polymerase Chain Reaction (PCR) for diagnosis of *P.vivax*

	Parascreen	Falcivax	Malascan	First Response	ParaHIT Total
**Light Microscopy as Gold Standard (Reference)**					

Sensitivity	77.2	68.4	68.4	84.2	15.8
(95% CI)	(64.2-87.3)	(54.8-80.1)	(54.8-80.1)	(72.1-92.5)	(7.5-27.9)
Specificity	98.1	99.0	97.8	96.5	100.0
(95% CI)	(95.9-99.3)	(97.2-99.8)	(95.5-99.1)	(93.8-98.2)	(98.8-100.0)
Positive Likelihood Ratio	40.5	71.8	30.8	24.1	
(95% CI)	(18.1-90.6)	(23.0-224.6)	(14.5-65.4)	(13.4-43.6)	
Negative Likelihood Ratio	0.23	0.32	0.32	0.16	0.84
(95% CI)	(0.14-0.37)	(0.22-0.47)	(0.22-0.47)	(0.09-0.30)	(0.75-0.94)
Positive Predictive Value	88.0	92.9	84.8	81.4	100.0
(95% CI)	(75.7-95.5)	(80.5-98.5)	(71.1-93.7)	(69.1-90.3)	(66.4-100.0)
Negative Predictive Value	96.0	94.5	94.5	97.1	86.8
(95% CI)	(93.2-97.8)	(91.5-96.7)	(91.4-96.7)	(94.6-98.7)	(82.9-90.1)
Percentage Agreement (Accuracy)	94.9	94.4	93.3	94.6	87.1
Kappa	0.80	0.76	0.72	0.80	0.24

**PCR as Gold Standard (Reference)**					

Sensitivity	68.3	61.7	63.3	76.7	13.3
(95% CI)	(55.0-79.7)	(48.2-73.9)	(49.9-75.4)	(64.0-86.6)	(5.9-24.6)
Specificity	97.0	98.4	97.4	95.7	99.7
(95% CI)	(94.5-98.6)	(96.2-99.5)	(94.9-98.9)	(92.8-97.7)	(98.2-100.0)
Positive Likelihood Ratio	23.1	37.5	24.1	17.9	40.5
(95% CI)	(11.9-44.9)	(15.4-91.5)	(11.8-49.0)	(10.4-31.1)	(5.2-318.1)
Negative Likelihood Ratio	0.33	0.39	0.38	0.24	0.87
(95% CI)	(0.22-0.47)	(0.28-0.54)	(0.27-0.53)	(0.15-0.39)	(0.79-0.96)
Positive Predictive Value	82.0	88.1	82.6	78.0	88.9
(95% CI)	(68.6-91.4)	(74.4-96.0)	(68.6.-92.2)	(65.3-87.7)	(51.8-99.7)
Negative Predictive Value	93.9	92.9	93.1	95.4	85.4
(95% CI)	(90.7-96.3)	(89.5-95.4)	(89.7-95.6)	(92.4-97.5)	(81.2-88.9)
Percentage Agreement (Accuracy)	92.3	92.3	91.8	92.6	85.4
Kappa	0.70	0.68	0.67	0.73	0.20

**Figure 4:**
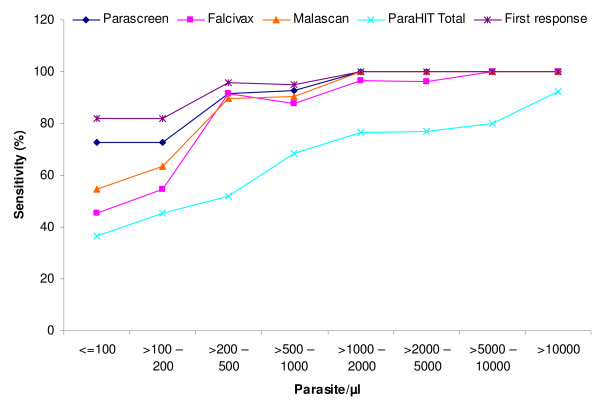
Showing sensitivity of five RDTs according to parasitaemia

The analysis of RDTs sensitivity according to parasitaemia revealed that the First Response was most sensitive for diagnosis of *P. falciparum *(95.0%) and *P. vivax *(88.0%) malaria as compared to other 4 RDTs especially for levels of parasitaemia above 200 parasite/μl (Figure 4). It should be noted that in this study because of the requirement for fever in patients from a high transmission area, there were no cases in which the parasite density was ≤ 40 parasites/μl.

**Figure 5:**
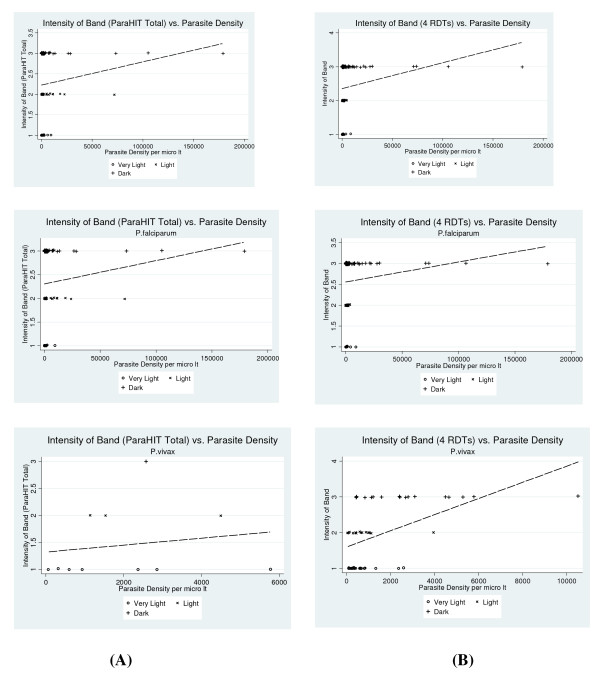
**Scatter plot showing association between intensity of band (A: ParaHIT Total; B: All other 4 RDTs) and Parasite density/μl**.

Analysis of intensity of band and parasite density revealed that there was a weaker correlation in band intensity and parasite density for ParaHIT Total r = 0.13 (P > 0.05). Although all other four RDTs was also showing a weak positive correlation in band intensity and parasite density but it was statistically significant r = 0.17 (P < 0.025). Species wise further analysis revealed that in *P. vivax *all four RDTs i.e. Parascreen, Falcivax, Malascan and First Response showed very strong positive correlation in band intensity and parasite density (r = 0.50, P < 0.0001) while no statistically significant correlation was seen in *P. falciparum *(Figure 5).

Further, exposure of all RDTs to high temperature i.e. 35°C, 45° & 60°C and low temperature (-10°C) did not cause any loss of sensitivity for both *P. falciparum *and *P. vivax *except ParaHIT Total when compared with microscopy and PCR. However, there was some reduction in test line-intensity at high temperature.

## Discussion

This comparative evaluation was carried out in outbreak-affected areas. From a malaria transmission perspective in both the areas, the RDTs can play a key role in rapid diagnosis and prompt treatment of malaria where resistance to CQ also necessitates the use of more expensive ACT. As RDT can be conducted immediately in the field clinic while the patient is present, the most important point for the villagers is the knowledge that they are infected with malaria parasite. On the contrary, the delay in the results of microscopic diagnosis is a serious obstacle for the operation of a malaria control programme in remote areas. Despite some inherent limitations, out of five tests evaluated, the First response was highly sensitive for the diagnosis of *P. falciparum *and non-falciparum especially for levels of parasitaemia above 200 parasite/μl. On the other hand its specificity was much lower than its sensitivity. Having a relatively low specificity which leads to an over-diagnosis and to an over treatment of non-malaria cases was, however, considered as less serious in such outbreak affected areas than having a low sensitivity which may lead to a potentially fatal condition being missed [24]. However, in a field setting such as ours, a negative RDT corresponds in the vast majority of cases to a non-infected individual. The high NPV allow us to confidently diagnose negative test patients as non-malaria patients [25]. Thus the risk of missing an infected individual is very small by most RDTs used in this evaluation. In Ethiopia high NPV was also recorded using Parascreen RDT in a population-based study [26] and in a health facility based study [27]. However, the sensitivity of all RDTs except First Response for non-*P. falciparum *infections is low (16-77%) as reported earlier from India using First Response and Falcivax RDTs [19,28].

The performance of RDTs can be adversely affected at the temperature to which they are exposed when transported [21]. Temperatures of 35°C to 45°C are common in malaria-endemic regions and higher temperatures may be encountered during transportation. Further inadvertent freezing has also been recorded during routine shipment [29]. All types of RDTs (except ParaHIT Total) perform satisfactorily at all temperatures although we do not know whether the performance of RDTs will be equally good at low parasitaemia as recorded by some investigators [21].

A diagnostic test which is to be used in a peripheral health facility, particularly in resource poor areas, has to be simple and fast to perform by less qualified staff. Among the five RDTs tested, First Response require 20 minutes while all other RDTs require 30 minutes before classifying the RDT as negative test. Further, the First Response need only 2 drop of buffer while all other RDTs need 4 drops. Thus the First Response clearly has an advantage over other RDTs.

## Conclusions

The two potential alternatives to microscopy are, PCR and RDTs. Primers exist for the reliable identification of the human malarias by PCR assays [22,30]. However, this is largely a research tool unsuited for routine use in the field or clinical laboratory. Given the logistic and financial difficulties of the PCR in field settings, only microscopy and RDTs are viable options at present and PCR remain a future alternative to these tests when inexpensive hand held diagnostic point of care (POC) instrumentation to detect malaria is available [31]. The practical and fast nature of RDTs make them the only currently viable supplement to or replacement of microscopy based diagnosis. Thus, RDTs could play and will play an important role in malaria diagnosis in the future. However, there are reservations about how well these RDTs perform as many commercially available RDTs lack the consistency, quality control and performance capabilities as claimed by the manufacturers making their use ineffective or potentially dangerous [32]. Further, can RDTs be operated by villagers, school-teachers or forest workers, in forested inaccessible areas so that they can penetrate into areas where microscope and health facilities are non existent? This is an important question which can only be answered when more experimental next generation RDTs are available.

## Competing interests

The authors declare that they have no competing interests.

## Authors' contributions

NS*: Study design, Data analysis and manuscript preparation. MMS: Clinical and field work. MKS: Field work and RDTs analysis. RKM: Field work and RDTs analysis. SS: Laboratory experiments. PKB: Laboratory experiments and PCR analysis. MPS: Data analysis, interpretation and manuscript preparation. AS: Data analysis and interpretation. AG: Study design, test interpretation and manuscript preparation. All authors read and approved the final manuscript.
